# Yin and yang roles of B lymphocytes in solid tumors: Balance between antitumor immunity and immune tolerance/immunosuppression in tumor-draining lymph nodes

**DOI:** 10.3389/fonc.2023.1088129

**Published:** 2023-01-24

**Authors:** Tomoya Katakai

**Affiliations:** Department of Immunology, Niigata University Graduate School of Medical and Dental Sciences, Niigata, Japan

**Keywords:** antitumor, B cells, immunotherapy, lymph node, protumor, regulatory lymphocytes, tolerance, tumor antigen

## Abstract

The role of B cells in antitumor immunity has been reported to be either promotive or suppressive, but the specific mechanism remains to be comprehensively understood. However, this complicated situation likely depends on the temporal and spatial relationship between the developing tumor and B cells that recognize tumor antigens. Unlike responses against microbial or pathogenic infections, tumor cells are derived from autologous cells that have mutated and become aberrant; thus, elimination by the adaptive immune system is essentially inefficient. If tumor cells can evade immune attack at an early stage, non-destructive responses, such as tolerance and immunosuppression, are established over time. In tumor-draining lymph nodes (TDLNs), tumor antigen-reactive B cells potentially acquire immunoregulatory phenotypes and contribute to an immunosuppressive microenvironment. Therefore, triggering and enhancing antitumor responses by immunotherapies require selective control of these regulatory B cell subsets in TDLNs. In contrast, B cell infiltration and formation of tertiary lymphoid structures in tumors are positively correlated with therapeutic prognosis, suggesting that tumor antigen-specific activation of B cells and antibody production are advantageous for antitumor immunity in mid- to late-stage tumors. Given that the presence of B cells in tumor tissues may reflect the ongoing antitumor response in TDLNs, therapeutic induction and enhancement of these lymphocytes are expected to increase the overall effectiveness of immunotherapy. Therefore, B cells are promising targets, but the spatiotemporal balance of the subsets that exhibit opposite characteristics, that is, the protumor or antitumor state in TDLNs, should be understood, and strategies to separately control their functions should be developed to maximize the clinical outcome.

## Introduction

Tumorigenesis and immunity are closely linked by nature (1–4). If the adaptive immune system can efficiently recognize genetic mutations in tumor cells as target antigens in the same way to eliminate foreign substances and pathogens, tumor development can be suppressed. In other words, if the antitumor response is properly triggered, the tumors can be eliminated before their appearance. However, the condition of the patient in whom the tumor has been clinically manifested indicates the outpacing of tumor growth over antitumor immunity, or unresponsiveness or suppression of the immune system for some reason. Therefore, the balance between the protumor and antitumor states in immunity may be a critical determinant of tumor progression or suppression.

Various immunotherapies to treat tumor by activating the immune system were attempted in the past without remarkable progress for a long time (5, 6). However, the situation has changed with the development of procedures to enhance the antitumor responses by inhibiting the immune checkpoint molecules, a system that negatively regulates immunity (7, 8). With this breakthrough, various improvements have been made and technical progress is underway (7, 9). However, the overall success rate of immune checkpoint blockade (ICB) therapy remains insufficient (7, 10–12), indicating that the fundamental principles have yet to be fully elucidated. Therefore, there is an urgent need to clarify these mechanisms to further improve the efficacy of immunotherapy.

B cells are crucial players in the adaptive immune system as antibody-producing lymphocytes (13), but also play other roles by presenting antigens to T cells and regulating immune responses *via* cytokine production (14, 15). There have been many conflicting reports on the roles of B cells in various solid tumors; protumor or antitumor (16–21). The reason is not readily interpretable, but roughly falls into two categories as follows:

**(A)** In transplantable mouse cancer models, the loss/removal of B cells or their functional inhibition suppresses the tumor growth (22–33). Thus, B cells exhibit tumor-promoting and immunosuppressive functions.
**(B)** In human clinical specimens, B cell infiltration and formation of lymphoid aggregates containing B cells in tumor tissues are positively correlated with the prognosis and therapeutic efficacy (20, 34–48). Therefore, B cells have tumor suppressive and antitumor functions.

They likely reflect the immunological phases in tumor progression: early and mid- to late stages of B cell function, respectively. In addition, the balance between immune tolerance/suppression and antitumor response in tumor-draining lymph nodes (TDLNs), where tumor antigens are delivered to induce adaptive immunity (49–54), is the key to understanding the conflicting roles of B cells in solid tumors. In this review, I briefly discuss the role of TDLNs and B cells in tumors, especially focusing on the relationship between immune tolerance and antitumor response, presenting a simple model for complex events.

## Induction of antigen-specific adaptive immune response in tissue-draining lymph nodes

Most adaptive immune responses against pathogens and foreign substances are induced in the lymph nodes (LNs), a secondary lymphoid organ incorporated into the lymphatic system that collects body fluid (lymph fluid) from peripheral tissues *via* afferent lymphatics (55–57). LNs play key roles as strategic sites in the immune system, where many immune cells, mainly lymphocytes, accumulate. Given that waste and foreign substances in lymph fluid are filtered by LNs, it is a rational anatomical site for collecting and detecting antigens from peripheral tissues to elicit an immune response (56). LNs are also rich in blood vessels, and lymphocytes circulating in the blood enter LNs *via* the specialized vascular structure, high endothelial venules (57, 58). Lymphocytes stay in LNs for a certain period and migrate in the interstitial space in search for antigens, but those that do not encounter antigens leave the organ *via* efferent lymphatic vessels and return to the bloodstream (57, 59). Lymphocytes repeat this cycle for recirculation, visit many LNs, and patrol throughout the body to search for antigens.

**Figure 1:**
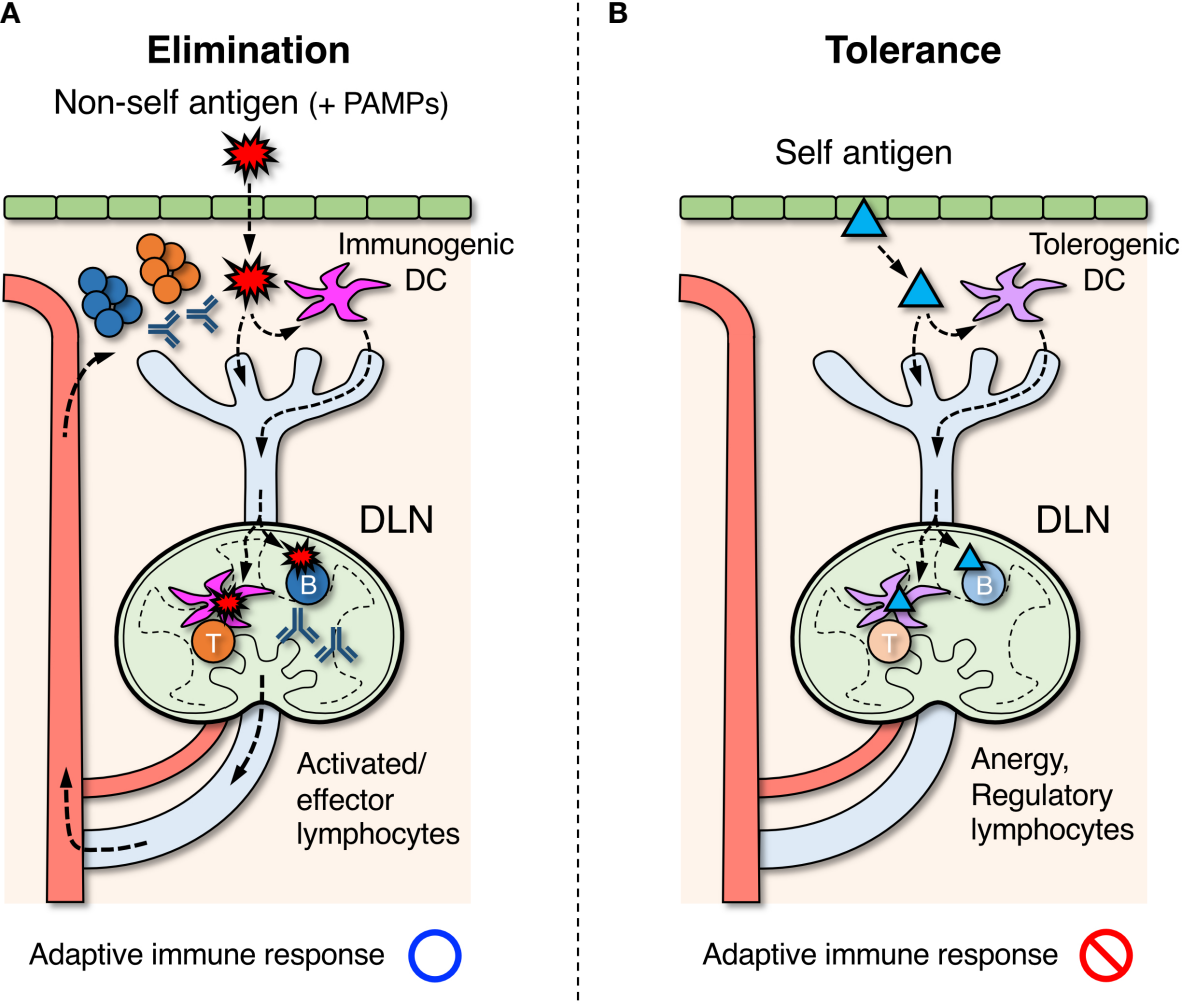
Roles of draining lymph nodes (DLNs) in the induction of adaptive immune response, elimination of foreign antigens, and tolerance to self-antigens. **(A)** Foreign antigens (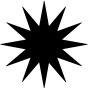) derived from microorganisms or pathogens that entered the body are taken up by dendritic cells (DCs) in peripheral tissues. If DCs are simultaneously stimulated by pathogen-associated molecular patterns (PAMPs), they acquire immunogenic property and migrate to DLNs *via* the lymphatic vessel. In DLNs, DCs further migrate into the paracortex to present antigens for activating T cells. Some of the antigens are also transported directly by lymph flow to the follicles of DLNs, and B cells that recognize them are activated. Activated lymphocytes are differentiated into effector cells and exit LNs *via* the efferent lymphatic vessel, returning to the blood circulation. They eventually infiltrate the site of inflammation and eliminate foreign antigens. **(B)** Self antigens (▴) in peripheral tissues are constitutively engulfed by DCs and transported to DLNs. In the absence of PAMP stimuli, DCs acquire tolerogenic property and induce unresponsive or regulatory function in self-reactive T cells in DLNs. Similarly, regulatory B cells are thought to be induced when self-reactive B cells recognize the same self-antigens.

The typical process to induce an adaptive response in LNs begins with the uptake of foreign antigens by dendritic cells (DCs) in peripheral tissues which move into the neighboring lymphatic vessels (**Figure 1A**) (60–62). During migration toward the tissue-draining LNs, DCs process engulfed antigens and present them on the cell surface as a peptide-MHC (pMHC) complex (63, 64). DCs that reach LNs migrate further into the paracortical T cell area and come into contact with numerous T cells. When antigen-specific T-cells recognize pMHC on DCs *via* the T-cell receptor (TCR), they are activated to proliferate and differentiate into effector cells (65). In contrast, some antigens that directly enter the lymphatic vessels and reach the same LNs are further transported into the follicular B-cell area *via* several pathways (66). Antigen-specific B cells are activated upon binding of antigens by the B-cell receptor (BCR) and uptake them to display pMHC (13, 14). If activated T cells detect the antigens presented on B cells, they support and facilitate further B cell activation to form the germinal center (GC) within the follicles (67). GC-B cells undergo somatic hypermutation in their immunoglobulin genes while proliferating aggressively within the GC, where B cells capable of producing antibodies with a higher affinity are selected. Immunoglobulin genes also undergo class-switch recombination, converting the constant region into a different subclass appropriate for the ongoing response. Activated and expanding lymphocytes then exit LNs *via* the efferent lymphatic vessel and migrate to the original inflammatory site to eradicate antigens. Most activated T and B cells die by apoptosis when the response converges, but some fractions survive for a long time as memory lymphocytes and prepare for the same antigen (68).

## Induction of peripheral self-tolerance

The immune system attacks and eliminates non-self-antigens derived from foreign microorganisms or pathogens, while it must establish tolerance and suppress responses to self-antigens of their own body components. In the primary lymphoid organs, thymus and bone marrow, central tolerance is formed by the elimination of lymphocyte clones that recognize the self-antigens during differentiation (69, 70). However, this process is imperfect and mature lymphocytes that are potentially reactive to self-components are inevitably generated at a certain frequency. These autoreactive clones are in turn suppressed by peripheral tolerance *via* the induction of anergy or active suppression by regulatory lymphocytes in secondary lymphoid organs (**Figure 1B**).

Anergy is the mechanism by which lymphocytes become unresponsive to cognate antigens in the absence of adequate co-stimuli by some molecular patterns of pathogens or cellular damages, pathogen-associated molecular patterns (PAMPs) or damage-associated molecular patterns (DAMPs), respectively (71–76). This means that even if lymphocytes that are reactive to self-antigens are present, tolerance can be achieved in the absence of infectious or damage-induced stimuli. Peripheral tolerance to self-antigens is induced in both T and B cells, albeit to different degrees and by different mechanisms (77). T cell unresponsiveness is mainly controlled by tolerogenic DCs (75, 78). Although the mechanism that induces B cell anergy/tolerance is not well understood, it is thought to be depending on some antigen structures or the lack of T cell help, i.e. T cell unresponsiveness (79, 80). Several studies have reported that anergic B cells show surface characteristics of B220^+^CD93^+^CD23^+^IgM^lo^ in mouse and IgD^+^IgM^lo/–^CD27^–^ in human (79, 81, 82).

Active suppression by regulatory cells occurs when immunosuppressive lymphocyte subsets are induced in primary and secondary lymphoid organs or possibly in inflammatory tissues (83, 84). Regulatory lymphocytes suppress immune responses using various mediators, such as cytokines, cell surface molecules, and metabolites. Although these cells differentiate under conditions similar to anergy induction, they appear to be derived from a fraction of cells that are highly responsive to self-antigens (85, 86). The best-known regulatory population is regulatory T (Treg) cells, including naturally occurring Tregs (nTregs) differentiated in the thymus and induced Tregs (iTregs) differentiated from naive T cells in peripheral tissues (83, 86). Recently, subsets of B cells with immunosuppressive functions have also been discovered and are collectively referred to as regulatory B (Breg) cells (21, 84, 87). The inhibitory effects of Breg cells on interleukin (IL)-10 production is well documented, although IL-10-independent processes have also been suggested. Some reports show that IL-10–producing Breg (B10) cells exhibit surface markers of CD19^+^CD5^+^CD1d^hi^ in mouse and CD19^+^CD24^hi^CD38^hi^ in human (21, 84, 88). These Breg cells may be important mediators of peripheral tolerance (84). Together, the above mechanisms for the induction and maintenance of self-tolerance in secondary lymphoid organs, such as LNs, likely inhibit the responses to newly emerging antigens.

## Induction of tumor antigen-specific adaptive immunity

**Figure 2:**
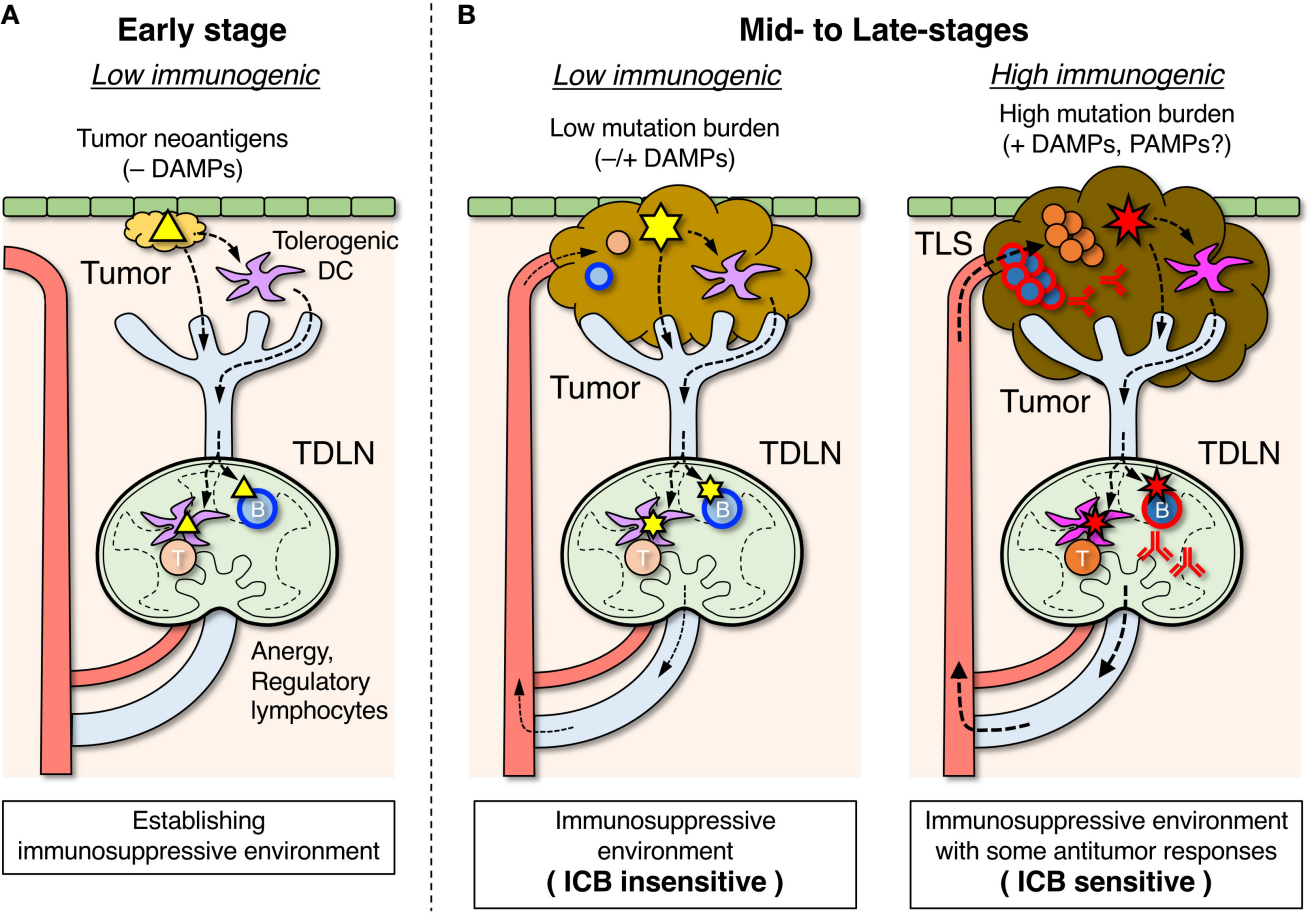
Roles of tumor-draining lymph nodes (TDLNs) in the suppression and activation of antitumor immune responses. **(A)** In the early stage, tumor antigens (△) are transported to TDLNs by DCs that have become tolerogenic in the absence of damage-associated molecular pattern (DAMP) stimuli and induce lymphocyte anergy and regulatory subsets which create an immunosuppressive environment. Some B cells differentiate into Breg cells, which are involved in suppressing the antitumor response. **(B)** In mid- to late-stages of tumor development, an immunosuppressive environment is already formed in TDLNs. When tumor mutation burden and DAMP stimuli are relatively low, emerging tumor antigens (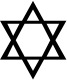) derived from new mutations are transported by tolerogenic DCs, and unresponsiveness of lymphocytes in TDLNs is maintained (left). When tumor mutation burden and DAMP stimuli due to immunogenic cell death are relatively high, the highly mutated tumor antigens (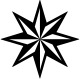) transported by immunogenic DCs can activate the antigen-specific lymphocytes in TDLNs to some degree (right), and a fraction of activated lymphocytes migrates to infiltrate the tumor or form tertiary lymphoid structures (TLSs). The efficacy of immune checkpoint blockade (ICB) therapy depends on the extent of the ongoing antitumor response.

Tumor cells originally emerged from autologous cells that acquired abnormal properties by genomic mutations (89). Changes in the protein sequence encoded by mutant genes create the possibility of recognition by the adaptive immune system as neoantigens (tumor antigens) (2, 90–92). These tumor antigens can be detected by lymphocytes in TDLNs, where lymph fluid drains from the primary tumor site (**Figure 2A**) (49–53). However, given that tumor cells in the early phase carry only a few mutations and differ little from normal cells, it is practically difficult for adaptive immunity to efficiently detect such rare tumor antigens. Moreover, in the absence of infection, lymphocyte clones that can recognize emerging antigens will inevitably become anergic and tolerized, as tumor cells are almost identical to normal cells. This may be the reason why, in many transplantable mouse tumor models, transfectant tumor cells that express foreign genes (models of newly generated tumor antigens) are tolerated by the host and are capable of growth.

Therefore, innate immunity that detects some cellular abnormalities other than antigen recognition plays a key role in the initial elimination of naturally occurring tumors (49, 93). However, tumor cells that show abnormalities undergo immunogenic cell death due to various cellular stresses and release DAMPs (94, 95). This potentially can lead to elicitation of adaptive immunity specific to tumor antigens (96). On the other hand, some tumor cells gradually gain the ability to suppress the immune system (49, 51, 97, 98). This creates an immunosuppressive microenvironment in the primary tumors or TDLNs, leading to accelerated tumor growth. Altogether, malignant tumors are those that have grown to become clinically apparent by evading various immune barriers and inhibiting the immune responses, making it difficult to be attacked by adaptive immunity.

## Role of TDLNs in the formation of an immunosuppressive environment

Similar to the induction of antitumor adaptive immunity, tumor antigen-specific immunosuppression/tolerance is also first developed in TDLNs (**Figure 2A**) (49–51, 54). In the absence of co-stimulation by infection or immunogenic cell death, anergic and regulatory lymphocytes may be induced by tumor antigens, as early cancer cells are almost identical to normal cells in terms of antigenicity. In such situations, DCs that have taken up tumor antigens in the primary tumors become biased toward immunosuppressive and tolerogenic properties owing to the absence of PAMP and DAMP stimuli and are involved in the induction of anergy and regulatory lymphocytes in TDLNs (78, 99). In addition, various immunosuppressive agents produced from tumor cells flow into TDLNs *via* lymphatic vessels, which also suppresses the antitumor response (97, 100, 101).

One of the key components responsible for forming an immunosuppressive environment is Treg cell induction. Several studies have shown an increase in Treg cells in TDLNs (54, 102–105). If tumor antigen-specific Treg cells are induced in TDLNs, cytotoxic T cells and other responses against tumor neoantigens that are normally induced may be limited (99, 106). These Treg cells in TDLNs eventually migrate to the tumor, contributing to the formation of a local immunosuppressive microenvironment (107).

Recent studies have indicated the importance of considering B-cell functions within TDLNs during tumor development (21, 87). Although the details remain unclear, tumor antigen-specific B cells in TDLNs are likely to acquire regulatory properties and contribute to the formation of an immunosuppressive environment. Tumor-derived soluble factors and cell fragments, such as exosomes containing neoantigens, are transported into the follicles of TDLNs, possibly inducing B-cell anergy/tolerance or Breg cells (100, 108–111). This event can proceed in parallel with the induction of tolerance in tumor antigen-specific T cells, and B cells that are incapable of receiving activation signals from helper T cells may become Breg cells.

## Protumor role of B cells: B cell-dependent tolerance/immunosuppression in TDLNs in early stage tumors

Several studies have suggested that B cells promote tumor progression in some situations, implying the suppression of antitumor immune responses (22–29, 31, 33). These findings are primarily based on the observation that the growth of transplanted tumor cells is significantly reduced in mice lacking B cells or their functions. In animal models in which tumor cells are transplantable to syngeneic hosts, tumor cells are tolerated by the immune system, probably because they are genetically almost identical to the host. However, changes that occur in TDLNs upon tumor formation will reflect, to some extent, the response to spontaneously developed tumors (112, 113). Inhibition of tumor growth is observed in B cell-deficient mice, such as µMT and JH^-/-^ mice, or by eliminating B cells *via* antibody administration (22, 24, 26, 28, 29, 31). In some cases, the loss of B cells enhances the influence of chemotherapy (23, 27, 30). These results suggest that the presence of B cells decreases the antitumor response. Notably, inhibition of tumor growth is also observed in BCR transgenic mice, such as MD4, in which the antigen specificity of B cells is fixed to a foreign antigen (28, 32). B cells in these mice lack diversity in terms of antigen specificity, meaning that tumor growth is inhibited in the absence of B cell reactivity to tumor antigens. This, in turn, indicates that immunosuppression by B cells is tumor-antigen-specific. It is assumed that inhibitory subsets, such as Breg cells, and their IL-10 production are important for the formation of an immunosuppressive environment (21, 87). Notably, recent reports showed that γ-aminobutyric acid (GABA) (31) and IL-35 (33) produced by B cells also exert protumor effects.

These studies suggest the induction of tolerance and/or regulatory subsets in tumor antigen-specific B cells in TDLNs during the early stages of tumor cell engraftment (**Figure 2A**). Lack of B cells or limited antigen specificity likely prevent the suppressive functions of B cells, leading to enhanced antitumor responses and decreased tumor growth. In fact, B cell-deficient mice show infiltration of cytotoxic T cells in tumors and increased cytolytic activity (24, 26, 27, 31). Therefore, it is speculated that during the early phase of tumor growth, B cells in TDLNs acquire immunosuppressive and protumor functions.

Since the absence of B cells promotes tumor growth in some mouse models (114, 115), B cells may exhibit antitumor effects depending on the tumor type or experimental condition. Moreover, genetic B cell deficiency may not properly represent the physiological role of B cells since the entire immune system is largely affected by the complete absence of B cells.

## Antitumor role of B cells: correlation between B-cell infiltration in tumors and the prognosis/treatment efficacy for mid- and late-stage tumors

Unlike inbred laboratory animals, which have a uniform genetic background and are maintained in a constant environment free of specific pathogens, humans are genetically and environmentally diverse, causing tumors to develop with varying age of onset, primary organ, subclassification/composition, and mutation burden. This results in a large set of clinical specimens or an epidemiological database with an assembly of countless different conditions that can be analyzed by focusing on a specific factor. Recently, remarkable progress has been made in the pathological examination of tumor tissues from large cohorts of clinical specimens, especially using computer-based analysis of digital images for the quantification of multiple parameters (116, 117). By coupling histopathological information with gene expression profiles, more powerful and multifaceted evaluations are possible (118).

Numerous histopathological analyses in a variety of solid tumors revealed that the degree of immune cell infiltration into tumor tissues, especially B cells and plasma cells (tumor-infiltrating B cells: TIBs), is significantly correlated with prognosis and therapeutic efficacy (18, 20, 34–48). The correlation with the efficiency of ICB therapy has attracted much attention (38, 40–42, 115, 119). TIBs include cells that exhibit characteristics of various differentiation stages and activation status, but activated or memory-like phenotypes are often abundant in association with a favorable prognosis and responsiveness to ICB therapy (18, 19, 35, 38, 40, 44, 45, 120, 121). Antibodies produced by B cells are possibly involved in the elimination of cancer cells *via* several mechanisms, suggesting that B cells can contribute to antitumor responses (19, 43, 47, 122, 123). B cells may also participate in tumoricidal responses through presentation of tumor antigens to T cells and antibody-dependent cytotoxicity in local tumor tissues. The presence of follicular helper T (Tfh) cells and the expression of C-X-C motif chemokine ligand 13 (CXCL13), a B cell-attracting chemokine, in tumor tissues are correlated with prognosis (18, 46, 124–127), suggesting that these are tightly linked with local B cell function.

Closely associated with TIBs in tumors, the formation of tertiary lymphoid structures (TLSs) or lymphocyte clusters within or adjacent to tumor tissues is correlated with prognosis and therapeutic efficacy (18, 40, 42, 45, 47, 118, 128, 129). TLSs are ectopic lymphocyte accumulations with tissue structures resembling LNs, which can be regarded as an aggregate of activated lymphocytes often induced by chronic inflammation (130). The formation of follicle-like structures with dense B cell accumulation is a remarkable indicator of TLSs; thus, the presence of TLS is synonymous with large-scale B cell infiltration. In these organized lymphoid structures, an environment that facilitates antitumor immunity, including B cell activation, antibody production, and activation of cytotoxic T cells *via* tumor-antigen presentation is formed (118, 129).

In addition to TIBs, active infiltration of other effector lymphocytes into the primary tumor should reflect an already present antitumor response in TDLNs (**Figure 2B**). Although insufficient to eliminate the tumor, the immune system is not completely suppressed, and a fraction of lymphocytes that have been activated in TDLNs can migrate and infiltrate the tumor. The differences in the intensity of lymphocyte infiltration are presumed to vary depending on genetic diversity and environmental factors (4, 92); the greater the lymphocyte infiltration into the tumor, the stronger is the ongoing antitumor response. Thus, it makes sense that lymphocyte infiltration in tumors is closely related to prognosis and response to ICB therapy. If a stronger antitumor response is already present, the patient would be more sensitive to ICB therapy due to the efficient activation of effector cytotoxic T cells, resulting in tumor suppression.

Taken together, the infiltration of B cells into tumors is a good indicator of ongoing antitumor immunity. If the balance between tolerance/immunosuppression and antitumor response in TDLNs is skewed toward the antitumor side, the degree of B cell tolerance/regulatory axis is weakened, resulting in more activated B cells entering the tumor lesion. If B cell activation or antibody production is linked to tumor suppression, it may directly correlate with prognosis after ICB therapy.

## Concluding remarks

Considering the dynamic behavior of B cells in TDLNs and tumor sites, the seemingly conflicting roles of B cells in various tumors can be regarded as reflections of the different phases of tumor progression as follows:

**(A)** Protumor/immunoregulatory B cell functions are linked to the induction of tolerance and an immunosuppressive environment in early stage TDLNs.
**(B)** Antitumor/tumor-suppressive B cell functions are linked to the degree of antitumor immune response in mid- and late-stage TDLNs.

In the protumor functions of B cells as **(A)**, the findings from genetically engineered animal models, in which the genetic background and environmental factors are homogeneous, represent the specific function of B cells in certain tumors. At present, it is difficult and risky to simply apply this knowledge to diverse human population for diagnostic and therapeutic purposes, such as for systemic B cell depletion. However, since B cells seem to contribute to the establishment of an immunosuppressive environment, it may be useful to combine ICB therapy with specifically targeting immunoregulatory B cells in the future.

The antitumor role of B cells **(B)** is extremely useful for diagnosis, therapeutic decisions, and prognosis prediction based on biopsies from patients, especially as promising information for the choice of immunotherapy, such as ICB. However, patients with low infiltration of B cells and other lymphocytes into the tumor have not yet overcome their insensitivity to immunotherapy. Various novel strategies currently underway to activate the antitumor immunity will provide more insights (9).

In any case, it should be noted that TDLNs play critical roles. TDLNs act as a reservoir of activatable lymphocytes that can potentially become effectors of antitumor response, making them a critical target for ICB therapy (110, 131–138). Therefore, careful decision is required for the removal of TDLNs or LN dissection. In addition, it is important to develop a rapid and simple method for determining the immune status of TDLNs in advance. The key point is how to appropriately restore or reactivate TDLNs from an immunosuppressive state. In the presence of TDLNs, the induction of immunogenic cell death in tumors, in addition to ICB therapy, may be the most effective (1, 95, 138–140). Manipulations that locally affect the primary tumors and TDLNs, e.g. intratumor or topical administration or treatment of therapeutic agents, are promising strategies for reducing the systemic adverse effects, while maximizing the therapeutic efficiency (132, 133, 138, 140–142). More detailed understanding of the spatiotemporal behavior of B cells in the context of the primary tumor–TDLN axis is required.

## Author contributions

The author confirms being the sole contributor of this work and has approved it for publication.
